# Perfluorinated alkyl substances in serum of the southern Chinese general population and potential impact on thyroid hormones

**DOI:** 10.1038/srep43380

**Published:** 2017-02-27

**Authors:** Yangjie Li, Yating Cheng, Zhiyong Xie, Feng Zeng

**Affiliations:** 1 School of Chemistry, Sun Yat-sen University, Guangdong, Guangzhou 510275, China; 2 Guangdong Institute for Drug Control, Guangdong, Guangzhou 510180, China; 3 KingMed Center for Clinical Laboratory Co., Ltd, Guangzhou 510330, China; 4 Helmholtz‒Zentrum Geesthacht, Centre for Materials and Coastal Research, Max‒Planck‒Strasse 1, Geesthacht, 21502, Germany

## Abstract

In this study, eight perfluorinated alkyl substances (PFASs) and five thyroid hormones (TSH, FT4, FT3, TGAb, and TMAb) were determined in 202 human serum samples of the general population of Guangdong, Guangxi and Hainan provinces in southern China. Σ_8_PFASs concentrations ranged from 0.85 to 24.3 ng/mL with a mean value of 4.66 ng/mL. The PFASs composition profiles of human serum samples nearly make no difference at different locations. A significant increase was observed for ∑_8_PFASs, PFOS, and PFHxS concentrations with age (p < 0.01). Gender-related differences were found; PFOS, PFHxS, PFBS, and PFOA levels were higher in males (p < 0.05), and the mean concentration of ∑_8_PFASs was 1.5 times greater in males (6.02 ng/mL) than in females (4.15 ng/mL). PFOS and ∑_8_PFASs were significantly negatively correlated with FT3 and FT4 and positively correlated with TSH while PFPeA and PFHxA were significantly positively correlated with TGAb and TMAb in all the samples. The opposite associations between FT3, TSH and PFOS, PFOA and PFHxS levels in hypothyroidism and hyperthyroidism group indicate that the PFOS, PFOA and PFHxS enhance the negative feedback mechanisms of the thyroid gland.

Perfluorinated alkyl substances (PFASs), which are characterized a strong carbon fluorine (C-F) covalent bonds that exhibit thermal and chemical stability, have been manufactured and used in a variety of industrial, commercial and consumer applications, such as water, oil, soil, and grease repellents in textiles and carpets, paper coatings, lubricants, alkaline cleaners, floor polish, and surfactants, since the 1950s^1^,^2^. In 2000, the 3 M Company voluntarily phased-out its production of perfluoro-octane sulfonyl fluoride (POSF)-based materials. However, China has been producing perfluoro-octanesulfonate (PFOS) & PFOS-related chemicals since 2003 and products made of perfluoro-octanyl-related materials are still widely used in China, especially in southern China, which is called the world’s factory due to its rapid economic growth, large economy and dense population of more than 100 million. Recent studies show that PFOS and perfluoro-octanoic acid (PFOA) levels in human blood from developed countries began to decline in 2000^3^,^4^,^5^, but the presence of PFASs remain of great concern due to their occurrence in the environment, human serum, breast milk and wildlife worldwide^6^,^7^,^8^,^9^,^10^,^11^. Many studies on PFASs exposure indicate that human exposure can be attributed to inhalation, dermal absorption and (mainly) diet, including drinking water and particularly fish consumption; these substances accumulate in the liver, kidney and blood by associating with proteins (but not typically with lipids)^12^,^13^,^14^,^15^,^16^,^17^,^18^. Generally, serum concentrations of PFASs lie in the μg/L range, and people in Western developed countries present higher concentrations than people in developing countries^9^. PFOS, PFOA, and perfluorohexanesulfonate (PFHxS) are usually the most frequently found PFASs and are found at higher levels^19^. The renal clearance of PFOA and PFOS is negligible in humans, leading to reported blood serum half-lives of 3.8 and 5.4 years for PFOA and PFOS, respectively^20^. Toxicological studies have demonstrated that PFOS and PFOA may have adverse effects in laboratory animals and on human health^19^,^21^,^22^,^23^,^24^. Due to its persistence, bioaccumulation and toxicity, PFOS has been added to the 2009 Stockholm Convention list of POPs.

Thyroid hormones play critical roles in many physiological processes, including growth, metabolism, and reproductive and nervous system functions^25^. TH is biological molecules that are halogenated by iodine, and PFASs are halogenated by fluorine with active sites. Thus, thyroid function is susceptible to disruption by PFASs compounds, which include the central control levels (thyrotropin-releasing hormone [TRH]/TSH), the levels of iodine uptake and hormone synthesis (thyroid gland), blood stream distribution, membrane based TH transporters or metabolism (conjugation or deiodination) to affect hormone homeostasis^26^. Numerous animal studies have shown that PFASs cause hypothyroxinemia in PFOS-exposed rats which is characterized by the depression of serum T4 and T3 without a concomitant increase in thyroid stimulating hormone (TSH) and a hypothyroid effect of PFASs in monkeys which is characterized by higher TSH and lower T4 and T3 levels^27^,^28^. However, the data on the effects of PFASs on human thyroid hormone balance are generally inconsistent; opposing positive, negative and null associations are found between various PFASs and human thyroid hormones^23^,^29^,^30^,^31^,^32^,^33^,^34^,^35^,^36^,^37^. In conclusion, these findings suggest that the effects of PFASs on thyroid hormone are still no general agreement.

The purposes of this study were as follows: (1) to investigate the exposure levels of eight PFASs in human serum samples obtained from the general populations of three provinces of southern China especially in the Guangdong province which is the most industrialized province of China, (2) by assessing associations between thyroid function and serum PFASs to illustrate whether PFASs impair thyroid hormone homeostasis in general populations of the economic developed area, and (3) to clarify whether the associations between perfluorinated alkyl substances and thyroid hormones are same in different thyroid diseases subgroups.

## Results

### Characteristics of the study participants

The characteristics of the study participants are summarized in Table 1 and Supplemental Table 1. The age of participants ranged between 1 month and 90 years old.

### PFASs and thyroid hormone concentrations in the serum samples

**Figure 1:**
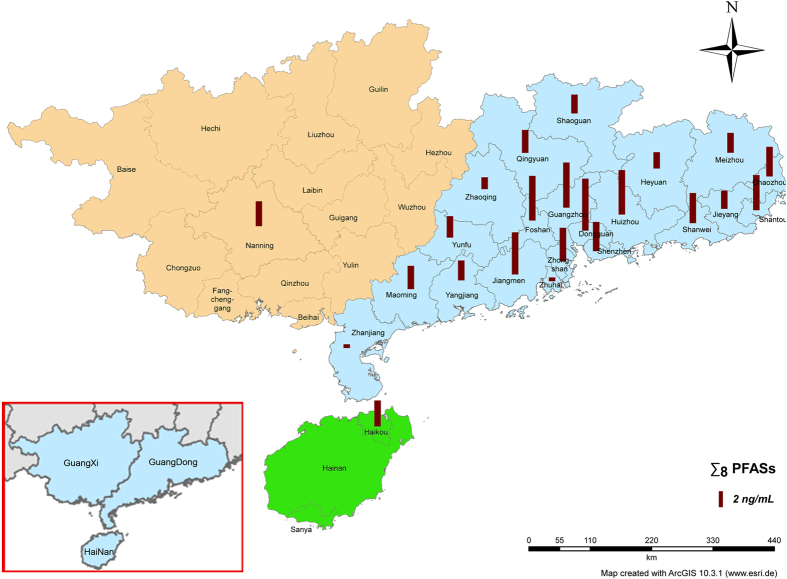


The mean and median concentrations of eight PFASs in the serum samples representing participants of different age, gender and thyroid hormones are summarized in Table 1. High frequencies of detection were obtained for PFOS (100.0%), PFOA (100.0%), PFHxS (98.0%), PFPeA (93.1%), PFPrA (91.1%), PFBA (75.2%), and PFHxA (53.0%). PFBS was detected in only 26.7% of the samples at concentrations near the LOQ. Σ_8_PFASs concentration in the serum samples ranged from 0.85 to 24.3 ng/mL (mean, 4.66 ng/mL). Among the target analytes, PFOS exhibited the highest mean concentration (2.01 ng/mL; range from 0.05 to 18.6 ng/mL) followed by PFOA (mean 1.78 ng/mL; range from 0.24 to 7.36 ng/mL). The geographical trends of PFASs that were found in serum samples obtained from three provinces of southern China are listed in Supplemental Table 1 and are marked on the map in different colors (Fig. 1: Guangxi, Hainan provinces and 19 cities of Guangdong province except Zhu hai and Zhan jiang city). Among the three provinces investigated in this study, the greatest Σ_8_PFASs concentrations were found in participants from Guangzhou (24.3 ng/mL). The greatest mean concentrations of Σ_8_PFASs were found in participants from Dongguan (6.86 ng/mL), which has been called the world’s factory, and the lowest mean concentrations of Σ_8_PFASs were found in participants from Zhaoqing (1.57 ng/mL). The greatest mean concentrations of PFOS and PFOA were found in participants from Foshan and Shantou, which are all in Guangdong province.

The reference ranges in euthyroid humans are 0.270–4.200 μIU/mL for TSH, 12.00–22.00 pmol/L for FT4, 3.10–6.80 pmol/L for FT3, 0.0–30.0% for TGAb and 0.0–20.0% for TMAb. Among the study population, median serum FT3, FT4, TSH, TGAb, and TMAb concentration were 4.5 pmol/L, 17.0 pmol/L, 1.1 uIU/mL, 36.0% and 22.7%. The values obtained for these five thyroid hormone parameters from different subclinical thyroid disease groups are listed in Supplemental Table 2.

### PFASs composition in the serum samples

**Figure 2:**
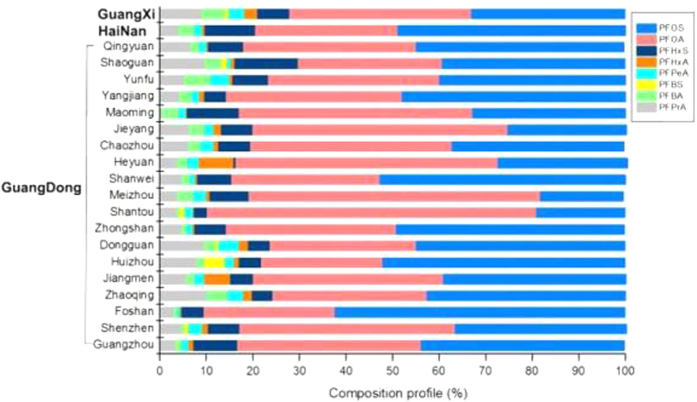


For all donors (n = 202), PFOS and PFOA were the dominant PFASs, contributing 42.8% and 38.5% of the mean concentration of ∑_8_PFASs (Supplemental Figure 1). Besides the sequence of PFOS and PFOA, the PFASs profiles of the serum samples make no difference at different locations (Fig. 2: Guangxi, Hainan provinces and 19 cities of Guangdong province); PFOS and PFOA were always the dominant PFASs, accounting for 70.3–90.5% of the ∑_8_PFASs. PFOS and PFOA are stable and persistent endproducts of POSF metabolism. The Pearl River Delta and the northern and western regions of Guangdong province (except for Maoming and Shenzhen cities) and Hainan province exhibited similar compositions. The greatest contribution in each city was from PFOS, ranging from 39.9 to 62.3%. The eastern region of Guangdong (except Shanwei city) and Guangxi provinces exhibited similar composition patterns, with PFOA contributing 40.9–62.6% of the total PFASs. The different composition profiles of the PFASs suggested that different exposure sources or pathways of PFASs might exist in the general population.

### Correlation between the environment, economical parameters and PFASs concentrations

Some parameters related to the environment and the economy (for example, the air, water and ecological environment quality index and GDP) are listed in Supplemental Table 3. The Spearman rank correlation coefficients between the environment, economical parameters and PFASs concentrations are listed in Table 2. The ecological environment index includes a series of comprehensive index including an organism abundance index, a vegetation index, a water network density index, a land degradation index and an environmental quality index and therefore reflects an evaluation of the regional ecological environmental quality. The Ecological Environment Index (EI) is inversely proportional to environmental quality. All the results of index are from department of environmental protection of Guangdong province. With the exception of PFBA, nearly all PFASs showed positive correlations with GDP; values of *r* ranged from 0.35 to 0.67. This finding indicates that economic development may enhance PFASs concentrations in human serum. A negative correlation was observed between the ecological environment index and the concentrations of PFOS (*r* = −0.40, *p* = 0.107), PFOA (*r* = −0.59, *p* = 0.013), PFPeA (*r* = −0.44, *p* = 0.081) and ∑_8_PFASs (*r* = −0.54, *p* = 0.026). The water quality index is more relevant than the air quality index, especially in relation to the concentrations of PFOS (WQD: *r* = 0.61, *p* = 0.01; AQI: *r* = 0.14, *p* > *0.05*) and PFHxS (*r* = 0.53, *p* = 0.03; *r* = 0.27, *p* > *0.05*). This comparison shows that drinking water is a more significant source of exposure than air especially to the PFOS and PFHxS.

### Potential impact on thyroid hormones of PFASs

Table 3 presents bivariate associations between PFASs and thyroid hormone levels according to thyroid disease classifications. As a general convention, associations with compounds detected in <70% of samples are suspect, and less than 50% detection is not appropriate, so PFBS was not listed in the Table 3 for only being detected in 27% samples. In the present study, PFOS (*p* < 0.01) and ∑_8_PFASs (*p* < 0.05) were significantly negatively correlated with FT3 and FT4 and positively correlated with TSH in all the samples (n = 202), consistent with the conclusion regarding fluorochemical production workers by Olsen and studies in monkeys by Butenhoff and in rats by Lau^19^,^20^,^28^. There are also consistent correlation between PFOA, PFPeA, PFBA and PFHxS and FT3, FT4 and TSH although not significant (n = 202). PFOS, PFOA and PFHxS were negatively correlated with FT4 in nearly all subgroups except normal group; however, more significant negative associations were observed in the hyperthyroidism group than hypothyroidism and other subgroups. In the hyperthyroidism group (n = 57), negative correlations were found between elevated FT3 and PFOS (*p* < 0.05), PFOA and PFHxS levels, whereas in the hypothyroidism group (n = 17), the opposite positive associations were observed between reduced FT3 and PFOS, PFOA and PFHxS levels. There are also opposite associations between TSH and PFOS, PFOA and PFHxS levels in hypothyroidism and hyperthyroidism group. PFPeA and PFHxA were significantly positively correlated with TGAb (*p* < 0.01) and TMAb (PFPeA: *p* < 0.05, PFHxA: *p* < 0.01) in all the samples (n = 202), however, in the Hashimoto’s disease group (n = 109) statistically significant associations between the levels of TGAb and TMAb with the concentrations of PFASs were no longer observed.

To assess the potential associations of serum PFASs with five thyroid hormones dependent variables adjusting for confounders such as age and sex, multivariable linear regression analyses were performed by stepwise method. The regression models included age and sex as covariates, and the data were log-transformed and assigned to quartiles according to PFASs concentration. Table 4 presents the associations between thyroid hormone levels and interquartile range increase in serum PFASs concentrations. Only eleven groups of linear regressions were selected after using the stepwise method with *p* < 0.05. Multivariable linear regression analyses indicated that interquartile range (IQR) increases of PFOS were associated with 2.4–16.1% decrease in FT3, 1.6–8.9% decrease in FT4 and 4.5–98.1% increase in TSH, whereas interquartile range (IQR) increases of PFHxA were associated with 10.4–33.5% increase in TGAb and 9.8–36.4% increase in TMAb when the sample was not stratified (n = 202). For the hyperthyroidism group (n = 57), multivariable linear regression analyses indicated that interquartile range (IQR) increases of ∑_8_PFASs were associated with 1.7–23.1% decrease in FT3 and 1.4–12.5% decrease in FT4, whereas interquartile range (IQR) increases of PFPeA was associated with 1.2–8.0% decrease in TSH. For the Hashimoto’s disease group (n = 109), multivariable linear regression analyses indicated that interquartile range (IQR) increases of PFOA were associated with 0.3–11.9% decrease in FT4.

## Discussion

The serum samples were stratified to clarify possible gender, age, region and thyroid hormone influences, respectively, on serum PFASs levels. The mean concentration of ∑_8_PFASs was 1.5 times greater in males (6.02 ng/mL, n = 55) than in females (4.15 ng/mL, n = 147) and was found to be significantly different (*p* < 0.01) between males and females. All three perfluoroalkyl sulfonates were also found at significantly greater concentrations in males than in females (*p* ranging from 0.00 to 0.03), suggesting that the pattern of PFSAs accumulation may be gender specific; however, the concentrations of PFCAs were not statistically different between the genders (*p* ranging from 0.09 to 0.92), with the exception of PFOA (*p* < 0.01). Heuvel *et al*. observed that female rats eliminated ^14^C-PFOA more rapidly than male rats (91% vs. 6%)^38^. Because PFASs accumulate in the blood by associating with proteins but are typically not associated with lipids, the blood clearance by menstruation every month in females is another important reason that can explain why males exhibit greater concentrations than females^39^.

For all donors (0–90 yrs), the mean concentration of ∑_8_PFASs was 2.7 times greater in the elders (5.65 ng/mL) than in infants (2.12 ng/mL), and PFOS (*r* = 0.29, *p* < 0.01), PFHxS (*r* = 0.34, *p* < 0.01) and ∑_8_PFASs (*r* = 0.23, *p* < 0.01) concentrations were also found to be significantly higher with age; however, no significant association was found between age and the concentrations of PFOA and the other five PFASs. The Spearmen rank correlation coefficients between age and PFASs concentrations are listed in Supplemental Table 4. The significant positive correlation between serum PFOS and PFHxS concentrations with age may be related to dietary intake, especially fish and seafoods that contain high levels of PFOS in China^40^. All the perfluorocarboxylic acids except PFPeA were found at greatest concentrations in infants and adolescents (Supplemental Fig. 2). The reasons for the higher PFCAs concentrations in infants and adolescents may due to different exposure sources and pathways, such as trans-placental transferring, breast milk, specific behaviors such as biting objects and ingesting dust while the contribution of dust ingestion to daily intake of PFOA was 3× higher in infants and adolescents than in adults^41^. These observations regarding gender and age are in general accord with most studies in other countries.

The Pearl River Delta is an important industrial manufacturing region in southern China, which produces electronic products, plastic products and textiles that are considered potential sources of PFASs. Moreover, as an important E-waste dismantling and recycling region in China, the soil, water, air and residents of Shantou are seriously exposed to environment pollution. Therefore, the Pearl River Delta region and Shantou show significantly greater (*p* = 0.004) concentrations of pollutants than other regions (Fig. 1 and Supplemental Table 1). The regional variation in serum PFASs levels might be due to differences in local contamination and the negative impact of economical development. The mean concentration of ∑_8_PFASs was 1.1 times greater in the abnormal thyroid hormone group (4.8 ng/mL, n = 140) than in normal group (4.4 ng/mL, n = 62), however, besides PFHxA (*p* = 0.000), no significant difference was found in concentrations of other PFASs and ∑_8_PFASs between normal and abnormal thyroid hormone group (*p* = 0.051–0.829).

After converting the concentrations of PFASs from values for whole blood of other studies to serum values by multiplying by a factor of 2, our data were compared with those from studies conducted in other countries (Supplemental Table 5). The mean concentrations of PFOS in serum samples from southern China were lower than those found in most studies and were comparable to those found in India and the Nanjing city of China, whereas the values for PFOA (mean, 1.78 ng/mL) were 1–3 times higher than those reported for the Hubei and Shanxi provinces of China and were approximately 1–2 orders of magnitude lower than those found in other countries (Korea: 123.6 ng/mL, United States: 3.73 ng/mL)^42^,^43^. In general, the human serum samples collected in southern China were slightly polluted by PFASs in comparison with the results found in other studies. Compared with other regions around the world, the contamination levels of PFASs in various environmental media of southern China, including the atmosphere, water, soil and seafood, were also relatively low^44^,^45^,^46^. To our knowledge, no fluorochemical manufacturing plant that might be a potential contaminant source of PFASs is present in the region examined in our study.

Analysis of individual PFASs is helpful for tracking contaminant sources and illustrating the fate and transport of PFASs in the environment and in bio-samples. Statistically, some PFASs analytes showed significant positive correlations (Supplemental Table 6 and Figure 3), suggesting a similar source of exposure, mechanism of elimination, or metabolic pathways. Significant positive correlations were observed between PFOS and PFOA (*r* = 0.51), PFOS and PFHxS (*r* = 0.65), PFOA and PFHxS (*r* = 0.53), PFPrA and PFBA (*r* = 0.51), and between PFPeA and PFHxA (*r* = 0.51). All of the above correlations were significant at the *p* = 0.01 level. PFOS is a major metabolite of POSF-based compounds, and PFHxS and PFOA are impurities in POSF-based products; this is likely why these 3 PFASs are correlated. As some PFASs were highly correlated, principal component analysis (PCA) was performed and PFOS, PFOA and PFHxS explained 34.5%, 24.6% and 12.5% of the total variance respectively (Supplemental Figure 3).

Thyroid function was assessed based on the levels of serum thyroid hormones (THs), such as TSH, FT4, FT3, TGAb and TMAb. FT3 and FT4 are specific index that indicates the clinical diagnosis of thyroid diseases such as hyperthyroidism (which is characterized by elevated FT3 and FT4 concentrations and reduced TSH concentrations) and hypothyroidism (which exhibits the opposite tendencies regarding FT3, FT4 and TSH concentrations). Thyroid binding globulin (TBG) is one of the most important serum binding protein in humans. TMAb and TGAb are marker of autoimmune hypothyroidism (Hashimoto’s disease), which occupied the highest proportion of thyroid diseases and characterized by elevated levels of TMAb and TGAb. The levels of thyroid hormones did not significantly differ between men and women and were not significantly correlated with age (data not shown). We analyzed associations between PFASs and thyroid hormones in the whole population (n = 202), and then in those subgroups with hyperthyroidism (n = 57), hypothyroidism (n = 17), Hashimoto’s disease (n = 109), and normal (n = 62) because prior studies suggest that susceptibility to PFASs may differ among these subgroups^47^. So the serum samples were stratified according to the value of five THs to clarify how the thyroid regulates THs levels to maintain equilibrium in the TH homeostasis and to clarify whether the associations between perfluorinated alkyl substances and thyroid hormones are same in different thyroid diseases subgroups.

T3 and T4 are iodinated biological molecules, whereas PFASs are halogenated by fluorine and have active sites. Thus, the thyroid is susceptible to disruption by PFASs-based compounds through the disturbance of hormone homeostasis by complex mechanisms such as interference with TH receptors, decreased activity of thyroperoxidase (TPO), which mediates iodine uptake by the thyroid gland for the synthesis of T3 and T4, and the competitive displacement of THs from their binding proteins^48^. Weiss have proven that PFOS can compete with T4 for binding sites on human transthyretin, leading to eventual decrease in thyroid hormone levels^49^. The decrease of thyroid hormone levels consequently increases TSH by compensatory mechanisms in the hypothalamus–pituitary–thyroid axis. Thyroid function is regulated by negative feedback mechanisms in which TSH stimulates the thyroid to synthesize T4, which is further converted to T3. T4 and T3 can combine with thyroid hormone-binding proteins (TBPs), including albumin and transthyretin, to transfer and regulate TH levels^50^. TSH is, in turn, regulated by the hypothalamus and by the levels of circulating T3 and T4 and is suppressed when more THs exist. TH serum levels are usually maintained at stable concentrations within the normal reference range. When THs concentrations change, the thyroid system accurately responds through negative regulations that naturally maintain TH equilibria. The opposite associations between FT3, TSH and PFOS, PFOA and PFHxS levels in hypothyroidism and hyperthyroidism group indicate that the PFOS, PFOA and PFHxS enhance the negative feedback mechanisms of the thyroid gland.

This study has several limitations. First, the recruited infants and adolescents were not representative of the lower age group population because the modest sample size (n = 12). Another limitation is the association should be interpreted with caution because of small sample sizes, especially in the hypothyroidism group (n = 17). Finally, PFASs accumulate in the blood by associating with proteins, so we should measure serum binding protein levels such as albumin and transthyretin. In light of the rapid development of the fluorine industry and the wide use of perfluorinated materials in China, further long-term monitoring should be conducted on the prospective impact of PFASs contamination on the population of China. The impact on the thyroid is particularly notable because these results were obtained in a general population with relatively low PFASs exposure.

## Materials and Methods

### Study participants and the collection of blood samples

Participants were restricted to general population instead of occupationally exposed workers. Because thyroid disease prevalence is markedly higher in women than in men, especially in the young adults, we recruited more than 50% young adults and 70% women to match reality of thyroid disease. We recruited 70% as the case group and 30% as the control group. Participants were restricted to have no thyroid medications. Further, in consideration of pregnancy and estrogen as potential influences with regard to thyroid function, pregnant, lactating women and who reported taking estrogen were excluded from the analytic sample. Thyroid disease was classified based on the measured thyroid hormones and clinical diagnosis. In a cross-sectional study involving an abnormal group (140 abnormal thyroid hormones) and a normal control group (62 normal thyroid hormones), participants of five age categories (the age classification standard of the World Health Organization; infants (0–1 yrs), adolescents (7–19 yrs), young adults (20–44 yrs), adults (45–59 yrs), and elders (60–90 yrs)) and both sexes (55 males and 147 females) were chosen in a local hospital, and their blood was collected between October 2013 and November 2014. All participants provided written informed consent before inclusion, and demographic factors including age, sex, location and date of collection were recorded. Details regarding the donors’ city of residence, age, and gender are provided in Table 1 and Supplemental Table 1. Cohort members were drawn from three provinces of southern China, including Guangdong, Guangxi and Hainan provinces. Guangdong province has become the first big economic province in China since 1989 and is famous for manufacturing industry. Guangxi province has the same dialects, customs, dietary habit and traditional culture but only 20% of GDP contrast to Guangdong province. Hainan province is an island of South China Sea and far away from terrigenous pollution, so was selected to be referential background.

Venous blood samples were drawn from each subject and centrifuged to obtain the upper serum layer. After routine clinical testing for thyroid hormones, the serum samples were stored in sanitized polypropylene cryovials at −80 °C until PFASs analysis. The present study was approved by the institutional review board of the School of Chemistry and Chemical Engineering, Sun Yat-Sen University (Guangzhou, China).

### Serum thyroid hormone determination

Serum thyroid hormones were measured at the KingMed Center Clinical laboratory, which has been accredited by China Association of Parliamentarians in Guangzhou, China. All methods were performed in accordance with the relevant guidelines and regulations for analysis thyroid hormones in human serum. Thyroid function was assessed by measuring the levels of TSH, free tetra-iodothyronine (FT4), free tri-iodothyronine (FT3), thyroglobulin antibodies (TGAb) and thyroid microsomal antibody (TMAb) in the serum samples. Concentrations of TSH, FT4, and FT3 in the serum samples were measured using an electro-chemiluminescence immunoassay (ECLIA, Roche 2010 system, Roche Diagnostics, U.S.A). TGAb and TMAb were measured based on the rate of antigen-antibody combination. The coefficients of variation (CV) for the TH assays were 2.1%, 5.3%, 4.0%, 4.5% and 8.1% for TSH, FT4, FT3, TGAb and TMAb, respectively.

### Reagents and materials

PFOS and PFOA blood levels are commonly used as biomarkers of human environmental exposure to these compounds. So we mainly choose eight short chained PFASs in our study. Eight target PFASs including three perfluoroalkyl sulfonates and five perfluorocarboxylic acids were analyzed in the serum samples: perfluorobutanesulfonate (PFBS), PFHxS, PFOS, pentafluoropropionic acid (PFPrA), perfluorobutyric acid (PFBA), perfluoropentanoic acid (PFPeA), perfluorohexanoic acid (PFHxA), and perfluoro-octanoic acid (PFOA) (Supplemental Table 7). ^13^C_4_- PFOA and ^13^C_4_- PFOS were used as internal standards (Wellington Laboratories), and control bovine serum was used to account for matrix effects. The organic solvents used in this experiment, including methyl tert-butyl ether (MTBE), methanol, acetonitrile, n-hexane and dichloromethane (DCM) were of HPLC grade and were obtained from the CNW Company. Anhydrous sodium sulfate, water and laboratory glassware were pretreated using standard laboratory procedures^51^.

### Sample extraction and instrumental analysis

PFASs in the serum samples were determined using the modified method developed by Hansen^52^. Briefly, each serum sample (1 mL) was spiked with 10 ng of ^13^C_4_-PFOA and ^13^C_4_-PFOS, and the mixture was then vortexed for 1 min with 20 μL formic acid to ensure homogeneity. After protein was precipitated using 3 mL of refrigerated acetonitrile, 0.5 g sodium sulfate, 2 mL DCM, 1 mL MTBE and 1 mL n-hexane were added to the tube, and the tube was then vortexed for 3 min. The solution was sonicated for 10 min and then centrifuged at 3000 × g for 10 min at 4 °C. The supernatant was removed to a second tube, and the remaining aqueous mixture was extracted again with 1 mL dichloromethane, 0.5 mL MTBE and 0.5 mL n-hexane. The two organic phases were pooled and concentrated using a vacuum concentrator, before reconstitution in 0.1 mL methanol. After vortexing for 30 s and centrifugation at 10000 × g for 10 mins at 4 °C, the supernatant was removed into an autosampler vial for LC/MS analysis.

PFASs were analyzed using high-performance liquid chromatography coupled with tandem mass spectrometry (HPLC-MS/MS). Chromatographic separation and quantification were performed using an Agilent Series 1260 Liquid Chromatograph system (Agilent Technologies, Santa Clara, CA), which was interfaced with an API 4500 triple-quadrupole mass spectrometer (Applied Biosystems/MDS-Sciex Instrument Corporation, Forest City, CA) operating in the electrospray ionization (ESI) negative mode with multiple reaction monitoring (MRM). The details of the instrument conditions used are listed in Supplemental and Supplemental Table 7.

### Quality Assurance and Quality Control (QA/QC)

To eliminate background contamination, fluoride-free glass centrifuge tubes were used instead of plastic tubes to extract the target objects. Quality control matrix recovery samples at three different spike levels (5, 20, 50 ng/mL) using control bovine serums in replicates of six (n = 6) were confirmed prior to actual sample analysis to ensure data accuracy and reliability. Newborn bovine serum has proven acceptable as a surrogate matrix for human sera in a thorough method validation^53^. Procedural blanks and matrix-spike recovery tests using quality control materials (QCs) that were associated with every one of the 14 serum unknowns were tested to check for possible laboratory contamination and measure recoveries. Only PFPeA, PFPrA and PFOA were detected at levels below 0.05 ng/mL in the procedure blanks, and the recoveries of all PFASs ranged between 86.1–122.0% in the matrix spikes. PFASs concentrations in the samples were not corrected for recovery but were corrected by subtraction of the background levels found in the procedure blanks. Method precision was excellent; the relative standard deviations of six analyses of the same blood samples were less than 10.0% for all eight PFASs. The intraday relative standard deviations (RSDs) (*n* = 6) of the spiked serum samples were between 3.3% and 8.0%. Target analyte concentrations were quantified using external calibration curves based on seven different concentrations (0.1, 1, 5, 10, 20, 50, and 100 ng/mL) and were calibrated using mass-labeled internal standards.

### Statistical analysis

Statistical analysis was performed using the statistics software package SPSS 19.0 (IBM SPSS Inc., Chicago, IL). For all statistical tests, any values below the LOQ were set to one-half of the LOQ, and any values below the LOD were assigned as values of the LOD/√2. The Kolmogorov-Smirnov test was used to validate whether the data satisfied the normal distribution. Because the PFASs concentrations were not normally distributed, we used the nonparametric Kruskal-Wallis or Mann-Whitney U tests to assess the significant differences measured. The Pearson correlation coefficient was applied to assess the relationships between the log-transformed PFASs concentrations and thyroid hormone parameters in the samples. The multivariable linear regression model (adjusting for potential confounders) was used to estimate associations of thyroid disease outcomes with PFASs concentrations. All statistical tests were two-tailed, and a *p*-value of 0.05 was chosen as the criterion for statistical significance in all analyses.

## Additional Information

**How to cite this article:** Li, Y. *et al*. Perfluorinated alkyl substances in serum of the southern Chinese general population and potential impact on thyroid hormones. *Sci. Rep.* **7**, 43380; doi: 10.1038/srep43380 (2017).

**Publisher's note:** Springer Nature remains neutral with regard to jurisdictional claims in published maps and institutional affiliations.

## Supplementary Material

Supplementary Information

## Figures and Tables

**Table 1:** Concentrations of PFASs (ng/mL) in serum samples from different age, genders and thyroid hormones groups.

		PFOS	PFOA	PFHxS	PFPrA	PFPeA	PFBA	PFHxA	PFBS	∑_8_PFASs
Total (n = 202)	Detection (%)^a^	100	100	98	91	93	75	53	27	
	median/mean	1.3/2.0	1.6/1.8	0.20/0.34	0.16/0.25	0.07/0.10	0.05/0.08	0.01/0.07	<LOD/0.03	3.8/4.7
	range	0.05–19	0.24–7.4	<LOD-2.3	<LOD-6.1	<LOD-2.8	<LOD-1.1	<LOD-1.1	<LOD-2.6	0.85–24
Sexes										
male (n = 55)	median/mean	1.8/2.7	2.2/2.2	0.49/0.60	0.16/0.23	0.06/0.09	0.03/0.07	0.02/0.10	<LOD/0.07	5.1/6.0
	range	0.08–19	0.28–4.9	<LOD-1.8	<LOD-1.7	<LOD-0.68	<LOD-0.46	<LOD-1.1	<LOD-2.6	1.2–24
female (n = 147)	median/mean	1.2/1.8	1.5/1.6	0.15/0.24	0.16/0.26	0.07/0.11	0.05/0.08	0.01/0.06	<LOD/0.01	3.6/4.2
	range	0.05–13	0.24–7.4	<LOD-2.3	<LOD-6.1	<LOD-2.8	<LOD-1.1	<LOD-0.90	<LOD-0.17	0.85–17
Age group^b^										
infants (n = 2)	median/mean	0.18/0.18	0.50/0.50	0.09/0.09	0.96/0.96	0.08/0.08	0.28/0.28	<LOD	0.02/0.02	2.1/2.1
	range	0.18–0.19	0.28–0.72	0.06–0.11	0.25–1.7	0.03–0.13	0.11–0.46	<LOD	<LOD-0.03	1.4–2.9
adolescents (n = 10)	median/mean	0.90/0.96	1.8/1.6	0.11/0.12	0.14/0.17	0.06/0.09	0.02/0.05	0.03/0.08	<LOD/0.01	3.0/3.1
	range	0.18–2.4	0.74–2.4	0.01–0.26	<LOD-0.48	<LOD-0.31	<LOD-0.24	<LOD-0.23	<LOD-0.05	1.3–5.5
young adults (n = 115)	median/mean	1.2/1.8	1.6/1.8	0.16/0.27	0.16/0.25	0.06/0.11	0.05/0.07	0.01/0.08	<LOD/0.04	3.6/4.4
	range	0.05–13	0.24–7.4	<LOD-1.8	<LOD-6.1	<LOD-2.8	<LOD-1.1	<LOD-1.1	<LOD-2.6	1.0–17
adults (n = 42)	median/mean	1.8/2.4	1.7/1.8	0.32/0.41	0.14/0.20	0.09/0.10	0.05/0.07	0.02/0.08	<LOD/0.01	4.3/5.0
	range	0.07–19	0.46–3.3	0.03–1.7	<LOD-1.7	<LOD-0.38	<LOD-0.29	<LOD-0.46	<LOD-0.13	0.85–24
elders (n = 33)	median/mean	2.0/2.7	1.7/1.9	0.42/0.57	0.17/0.28	0.06/0.08	0.08/0.09	<LOD/0.05	<LOD/0.02	4.7/5.7
	range	0.11–11	0.32–4.2	0.04–2.3	<LOD-1.5	<LOD-0.33	<LOD-0.43	<LOD-0.40	<LOD-0.15	0.90–15
Thyroid hormones										
normal (n = 62)	median/mean	1.4/2.0	1.5/1.6	0.24/0.33	0.17/0.24	0.06/0.07	0.05/0.06	0.01/0.03	<LOD/0.06	3.8/4.4
	range	0.07–11	0.24–3.8	<LOD-1.7	<LOD-1.7	<LOD-0.67	<LOD-0.46	<LOD-0.32	<LOD-2.6	0.85–15
abnormal (n = 140)	median/mean	1.3/2.0	1.6/1.9	0.19/0.34	0.15/0.25	0.07/0.11	0.05/0.08	0.03/0.09	<LOD/0.01	3.8/4.8
	range	0.05–19	0.32–7.4	<LOD-2.3	<LOD-6.1	<LOD-2.8	<LOD-1.1	<LOD-1.1	<LOD-0.2	0.90–24

^a^Detection = frequency of detection; ^b^Age group including infants (0–1 yrs), adolescents (7–19 yrs), young adults (20–44 yrs), adults (45–59 yrs), elders (60–90 yrs).

**Table 2:** Spearmen’s rank correlation coefficients between environment and economy parameters and PFASs concentrations.

PFAS	PM2.5^a^	PM2.5^b^	AQI^c^	WQD^d^	EI^e^	GDP^f^
PFOS	−0.021	0.068	0.14	0.606**	−0.405	0.539*
PFOA	0.069	0.178	−0.02	0.055	−0.59*	0.52*
PFHxS	0.141	−0.046	0.267	0.531*	−0.155	0.348
PFPrA	0.182	−0.072	0.256	0.409	−0.089	0.412
PFPeA	−0.053	0.233	−0.019	0.031	−0.435	0.369
PFBA	0.026	0.001	0.025	0.171	−0.025	−0.301
PFHxA	0.139	0.036	0.166	0.108	−0.069	0.346
PFBS	0.059	0.047	0.108	0.341	−0.151	0.671**
∑_8_PFASs	−0.064	0.206	0.04	0.442	−0.539	0.532*

^a^the mean concentrations of PM2.5 (μg/m^3^); ^b^the good rate of PM2.5 (%); ^c^Air Quality Index; ^d^water quality index; ^e^Ecological Environment Index; ^f^Gross Domestic Product (a hundred million yuan); **Correlation was significant at the 0.01 level; *Correlation was significant at the 0.05 level.

**Table 3:** Pearson correlation coefficients between serum thyroid hormones and PFASs concentrations in different thyroid disease group^a^.

		PFOS	PFOA	PFHxS	PFPrA	PFPeA	PFBA	PFHxA	∑_8_PFASs
Total (n = 202)	FT3	−0.189**	−0.059	−0.115	0.027	−0.051	−0.031	0.015	−0.153*
	FT4	−0.200**‘	−0.107	−0.099	0.032	−0.067	−0.053	−0.034	−0.180*
	TSH	0.158*	0.048	0.065	−0.042	0.013	0.027	−0.092	0.122
	TGAb	0.033	0.112	0.050	0.001	0.196**	0.043	0.273**	0.069
	TMAb	0.049	0.088	0.069	−0.013	0.170*	0.043	0.250**	0.065
Normal (n = 62)	FT3	0.186	0.072	0.175	−0.081	−0.203	−0.152	−0.232	0.095
	FT4	0.135	−0.047	0.038	−0.064	0.025	−0.190	−0.038	0.030
	TSH	−0.091	−0.186	−0.070	−0.197	−0.074	−0.348**	−0.273*	−0.163
	TGAb	−0.110	−0.126	−0.136	−0.034	0.084	−0.010	0.264*	−0.139
	TMAb	0.051	−0.219	0.024	−0.111	−0.060	−0.052	0.219	−0.096
Hyperthyroidism (n = 57)	FT3↑	−0.308*	−0.246	−0.236	−0.020	0.013	0.249	−0.128	−0.322*
	FT4↑	−0.300*	−0.297*	−0.210	0.013	−0.016	0.231	−0.185	−0.338*
	TSH↓	−0.092	−0.043	−0.237	−0.130	−0.350**	−0.237	−0.162	−0.014
	TGAb	0.123	0.185	0.068	0.112	0.232	0.017	0.160	0.171
	TMAb	0.096	0.152	0.068	0.109	0.207	0.033	0.101	0.144
Hypothyroidism (n = 17)	FT3↓	0.213	0.118	0.085	0.396	0.121	−0.365	0.114	0.233
	FT4↓	−0.196	−0.211	−0.195	0.025	−0.168	−0.259	−0.059	−0.200
	TSH↑	0.139	0.215	0.169	0.217	0.355	0.250	0.130	0.199
	TGAb	−0.355	−0.011	−0.021	0.092	0.220	0.435	−0.042	−0.236
	TMAb	−0.381	−0.051	−0.070	0.075	0.190	0.440	−0.078	−0.270
Hashimoto’s disease (n = 109)	FT3	−0.177	−0.157	−0.137	0.089	−0.090	−0.060	−0.044	−0.175
	FT4	−0.173	−0.199*	−0.070	0.083	−0.095	−0.075	−0.091	−0.188
	TSH	0.154	0.134	0.076	−0.053	0.030	0.081	−0.049	0.148
	TGAb↑	0.026	−0.005	0.026	−0.016	0.036	−0.003	0.082	0.057
	TMAb↑	−0.013	−0.027	0.008	−0.015	0.053	0.032	0.109	0.023

^a^log-transformed; **Correlation was significant at the 0.01 level; *Correlation was significant at the 0.05 level; ↑ means above the normal value; ↓ means below the normal value.

**Table 4:** Multivariable linear regression models for thyroid hormones with serum PFASs^a^.

	Thyroid hormone	PFASs	β	95% CI	p
Total (n = 202)	FT3	PFOS	−0.138	(−0.238, −0.038)	0.007
	FT4	PFOS	−0.131	(−0.220, −0.042)	0.004
	TSH	PFOS	0.406	(0.053, 0.759)	0.024
	TGAb	PFHxA	0.284	(0.144, 0.423)	0.000
	TMAb	PFHxA	0.253	(0.116, 0.390)	0.000
Normal (n = 62)	TSH	PFBA	−0.280	(−0.474, −0.085)	0.006
	TGAb	PFHxA	0.166	(0.009, 0.322)	0.038
Hyperthyroidism (n = 57)	FT3	∑_8_PFASs	−0.351	(−0.630, −0.072)	0.015
	FT4	∑_8_PFASs	−0.293	(−0.514, −0.073)	0.010
	TSH	PFPeA	−0.169	(−0.291, −0.046)	0.008
Hashimoto’s disease (n = 109)	FT4	PFOA	−0.255	(−0.496, −0.014)	0.038

*a*other PFASs and THs were not listed in the table for P > 0.05 after using stepwise method.
