# Supplementary material for: Perfluorinated alkyl substances in serum of the southern Chinese general population and potential impact on thyroid hormones
Source: Sci Rep. 2017 Feb 27;7:43380. doi: 10.1038/srep43380 (PMC5327476; doi:10.1038/srep43380)
Supplement: Supplementary Information [file srep43380-s1.doc]

**Supplemental information and Table**

**(13 Pages)**

**Perfluorinated alkyl substances in serum of the southern Chinese general population and potential impact on thyroid hormones**

**Yangjie Li ^1, 2^, Yating Cheng ^3^,** **Zhiyong Xie ^4, *^, and Feng Zeng ^1, *^**

^1^ School of Chemistry and Chemical Engineering, Sun Yat-sen University, Guangdong, Guangzhou 510275, China

^2^ Guangdong Institute for Drug Control, Guangdong, Guangzhou 510180, China

^3^ KingMed Center for Clinical Laboratory Co., Ltd, Guangzhou 510330, China

^4^ Helmholtz‒Zentrum Geesthacht, Centre for Materials and Coastal Research, Max‒ Planck ‒ Strasse 1, Geesthacht, 21502, Germany

***Corresponding Author**

Feng Zeng

Phone/fax: 86-20-84114133, E-mail: ceszf@mail.sysu.edu.cn

Zhiyong Xie

Phone: +49 4152872330, fax: +49 4152 872332, E-mail: zhiyong.xie@gkss.de

**Table of Content**

**S3:** Table 1. Geographical trends of mean PFASs (ng/mL) in serum samples from south China

**S4:** Table 2. The value of five thyroid hormone [parameters](javascript:void(0);) of serums from different

thyroid hormones groups

**S5:** Table 3. Environment, population and economy parameters and the mean concentrations of PFASs (ng/mL) from cities of Guangdong provinces

**S6:** Table 4. Spearmen’s rank correlation coefficients between age and PFASs

concentrations

**S7:** Table 5. Perfluorochemical mean concentrations in serum (ng/mL) from various

countries

**S8**: Table 6. Spearmen’s Rank Correlation Coefficients between PFASs

Concentrations

**S9:** Table 7. HPLC-MS-MS paramaters and information of PFASs and Instrument

conditions

**S10:** Figure1. Composition profiles of PFASs in serum from the southern Chinese

general population

**S11:** Figure 2. Line chart of the mean concentrations of PFASs in different age group.

**S12:** Figure 3. Plot of factors of principal component analysis of PFASs in serums among the southern Chinese general population

**S13:** Reference

| **Province** | **City** | **PFOS** | **PFOA** | **PFHxS** | **PFPrA** | **PFPeA** | **PFBA** | **PFHxA** | **PFBS** | **∑_8_PFASs** |
| --- | --- | --- | --- | --- | --- | --- | --- | --- | --- | --- |
| Guang dong province  (n=181) | Pearl River Delta (n=126) | | | | | | | | | |
|  | Guangzhou | 2.60 | 2.35 | 0.56 | 0.20 | 0.10 | 0.06 | 0.06 | 0.01 | 5.95 |
|  | Shenzhen | 1.42 | 1.79 | 0.26 | 0.18 | 0.11 | 0.03 | 0.05 | 0.03 | 3.86 |
|  | Foshan | 3.67 | 1.66 | 0.28 | 0.18 | 0.04 | 0.03 | 0.02 | 0.006 | 5.89 |
|  | Zhaoqing | 0.67 | 0.52 | 0.07 | 0.16 | 0.05 | 0.07 | 0.03 | 0.001 | 1.57 |
|  | Jiangmen | 2.17 | 2.27 | 0.27 | 0.32 | 0.12 | 0.08 | 0.31 | 0.01 | 5.55 |
|  | Huizhou | 3.07 | 1.53 | 0.28 | 0.47 | 0.12 | 0.10 | 0.06 | 0.25 | 5.88 |
|  | Dongguan | 3.09 | 2.16 | 0.32 | 0.66 | 0.30 | 0.17 | 0.13 | 0.04 | 6.86 |
|  | Zhongshan | 2.18 | 1.62 | 0.30 | 0.21 | 0.05 | 0.05 | 0.02 | 0.001 | 4.44 |
|  | Eastern Guangdong (n=30) | | | | | | | | | |
|  | Shantou | 0.89 | 3.29 | 0.13 | 0.17 | 0.08 | 0.03 | 0.01 | 0.05 | 4.65 |
|  | Meizhou | 0.47 | 1.64 | 0.22 | 0.10 | 0.07 | 0.09 | 0.02 | 0.001 | 2.62 |
|  | Shanwei | 2.10 | 1.27 | 0.29 | 0.19 | 0.04 | 0.07 | 0.02 | 0.001 | 3.98 |
|  | Heyuan | 0.61 | 1.21 | 0.01 | 0.08 | 0.05 | 0.05 | 0.16 | 0.001 | 2.15 |
|  | Chaozhou | 1.45 | 1.69 | 0.27 | 0.24 | 0.11 | 0.10 | 0.04 | 0.001 | 3.91 |
|  | Jieyang | 0.61 | 1.31 | 0.16 | 0.15 | 0.05 | 0.07 | 0.04 | 0.01 | 2.39 |
|  | Western Guangdong (n=10) | | | | | | | | | |
|  | Maoming | 1.01 | 1.54 | 0.34 | 0.02 | 0.05 | 0.1 | 0.01 | 0.001 | 3.07 |
|  | Yangjiang | 1.26 | 0.99 | 0.12 | 0.11 | 0.04 | 0.07 | 0.03 | 0.002 | 2.62 |
|  | Yunfu | 1.13 | 1.04 | 0.22 | 0.15 | 0.11 | 0.16 | 0.02 | 0.001 | 2.83 |
|  | Northern Guangdong (n=15) | | | | | | | | | |
|  | Shaoguan | 0.98 | 0.77 | 0.34 | 0.24 | 0.02 | 0.09 | 0.02 | 0.03 | 2.49 |
|  | Qingyuan | 1.36 | 1.12 | 0.23 | 0.20 | 0.05 | 0.05 | 0.01 | 0.003 | 3.02 |
| Hainan province (n=7) | | 1.68 | 1.05 | 0.37 | 0.14 | 0.06 | 0.11 | 0.02 | 0.002 | 3.43 |
| Guangxi province (n=14) | | 1.09 | 1.29 | 0.23 | 0.30 | 0.11 | 0.16 | 0.09 | 0.03 | 3.30 |

**Table S1.** Geographical Trends of mean PFASs (ng/mL) in Serum Samples from south China

|  |  | **FT3(pmol/L)** | **FT4 (pmol/L)** | **TSH (uIU/mL)** | **TGAb (%)** | **TMAb (%)** |
| --- | --- | --- | --- | --- | --- | --- |
| [normal](javascript:void(0);)  [range](javascript:void(0);) |  | 3.10-6.80 | 12.00-22.00 | 0.270-4.200 | 0.0-30.0 | 0.0-20.0 |
| Total (n=202) | median(mean) range | 4.53 (8.91)  2.03-50.0 | 17.03 (27.39) 4.39-100.0 | 1.07 (3.45)  0.01-100.0 | 36.0 (33.08)  1.20-77.80 | 22.7 (21.43) 0.90-50.70 |
| abnormal^a^ | median(mean) range | 2.69 (2.60) 2.03-3.07  (n=8) | 10.43 (9.84) 4.39-11.93  (n=26) | 0.01 (0.02) 0.01-0.18  (n=70) | / | / |
| control | median(mean) range | 4.31 (4.39) 3.14-6.75  (n=142) | 16.46 (16.43) 12.06-21.99  (n=117) | 1.60 (1.817) 0.28-3.93  (n=111) | 4.20 (6.85) 1.20-29.40  (n=92) | 2.80 (5.04) 0.90-19.7  (n=94) |
| abnormal^b^ | median(mean) range | 16.24 (22.2) 6.88-50.00  (n=52) | 44.84 (56.84) 22.36-100.0  (n=59) | 13.43 (23.53) 4.42-100.0  (n=21) | 55.50 (55.02) 30.20-77.80  (n=110) | 36.10 (35.69) 20.3-50.7  (n=108) |

**Table S2.** The value of five thyroid hormone [parameters](javascript:void(0);) of serums from different thyroid hormones groups. a:the value less than normal; b: the value greater than normal

| **City** | **PM2.5^b^** | **PM2.5^c^** | **AQI^d^** | **WQD^e^** | **EI^f^** | **GDP^g^** | **Population^h^** | **PFOS** | **PFOA** | **PFHxS** | **PFPrA** | **PFPeA** | **PFBA** | **PFHxA** | **∑_8_PFASs** |
| --- | --- | --- | --- | --- | --- | --- | --- | --- | --- | --- | --- | --- | --- | --- | --- |
| Chaozhou | 49 | 88 | 4.49 | 14 | 69 | 815 | 27.2 | 1.451 | 1.687 | 0.272 | 0.238 | 0.115 | 0.104 | 0.041 | 3.909 |
| Dongguan | 45 | 91 | 5.01 | 26 | 61 | 5686 | 83.4 | 3.086 | 2.164 | 0.323 | 0.659 | 0.299 | 0.170 | 0.127 | 6.864 |
| Foshan | 47 | 85 | 5.27 | 24 | 59 | 7307 | 73.5 | 3.668 | 1.661 | 0.285 | 0.176 | 0.044 | 0.030 | 0.016 | 5.888 |
| Guangzhou | 49 | 88 | 5.20 | 28 | 63 | 16064 | 13.1 | 2.605 | 2.353 | 0.563 | 0.204 | 0.101 | 0.055 | 0.057 | 5.951 |
| Heyuan | 39 | 94 | 4.07 | 11 | 78 | 723 | 30.6 | 0.605 | 1.206 | 0.010 | 0.079 | 0.045 | 0.046 | 0.158 | 2.150 |
| Huizhou | 35 | 97 | 3.97 | 17 | 78 | 2840 | 47.3 | 3.069 | 1.532 | 0.277 | 0.475 | 0.118 | 0.101 | 0.056 | 5.882 |
| Jiangmen | 44 | 86 | 4.63 | 22 | 72 | 2042 | 45.1 | 2.175 | 2.275 | 0.268 | 0.322 | 0.120 | 0.077 | 0.306 | 5.554 |
| Jieyang | 51 | 85 | 4.85 | 19 | 72 | 1693 | 60.4 | 0.614 | 1.311 | 0.156 | 0.148 | 0.049 | 0.068 | 0.041 | 2.393 |
| Meizhou | 40 | 93 | 4.03 | 14 | 77 | 843 | 43.2 | 0.466 | 1.644 | 0.223 | 0.105 | 0.070 | 0.090 | 0.025 | 2.624 |
| Qingyuan | 42 | 87 | 4.63 | 20 | 78 | 1141 | 38.2 | 1.357 | 1.117 | 0.231 | 0.201 | 0.051 | 0.049 | 0.010 | 3.019 |
| Shantou | 41 | 94 | 3.91 | 13 | 68 | 1641 | 55.2 | 0.891 | 3.292 | 0.134 | 0.166 | 0.076 | 0.031 | 0.010 | 4.653 |
| Shanwei | 34 | 97 | 3.34 | 16 | 78 | 694 | 30.1 | 2.100 | 1.273 | 0.295 | 0.187 | 0.039 | 0.066 | 0.017 | 3.977 |
| Shaoguan | 48 | 85 | 5.10 | 20 | 79 | 1061 | 29.1 | 0.984 | 0.775 | 0.338 | 0.243 | 0.015 | 0.087 | 0.020 | 2.495 |
| Shenzhen | 34 | 97 | 3.88 | 15 | 73 | 15251 | 107.8 | 1.416 | 1.787 | 0.264 | 0.184 | 0.112 | 0.026 | 0.047 | 3.865 |
| Yangjiang | 37 | 93 | 3.60 | 25 | 76 | 1105 | 25.0 | 1.260 | 0.993 | 0.122 | 0.105 | 0.044 | 0.070 | 0.027 | 2.622 |
| Yunfu | 32 | 96 | 3.94 | 19 | 67 | 633 | 24.4 | 1.126 | 1.038 | 0.218 | 0.154 | 0.110 | 0.163 | 0.017 | 2.827 |
| Zhaoqing | 52 | 81 | 5.28 | 15 | 74 | 1753 | 40.4 | 0.672 | 0.518 | 0.067 | 0.160 | 0.051 | 0.067 | 0.032 | 1.568 |

**Table S3.** Environment, population and economy parameters^a^ and the mean concentrations of PFASs (ng/mL) from Cities of Guangdong provinces. a:all the environment and economy parameters derived from government departments; b:the mean concentrations of PM2.5 (μg/m3); c:the good rate of PM2.5 (%); d:Air Quality Index; e:water quality index; f:Ecological Environment Index; g:Gross Domestic Product (a hundred million yuan); h:population(per 100,000)

|  | **both sex** | **male** | **female** |
| --- | --- | --- | --- |
| PFOS | 0.291** | 0.317* | 0.235** |
| PFOA | 0.037 | 0.098 | -0.032 |
| PFHxS | 0.344** | 0.375** | 0.279** |
| PFPrA | 0.034 | -0.048 | 0.033 |
| PFPeA | 0.029 | -0.017 | 0.053 |
| PFBA | 0.109 | 0.142 | 0.097 |
| PFHxA | 0.025 | -0.102 | 0.066 |
| PFBS | -0.108 | -0.204 | -0.094 |
| ∑_8_PFASs | 0.227** | 0.242 | 0.172* |

**Table S4.** Spearmen’s rank correlation coefficients between age and PFASs concentrations. **Correlation was significant at the 0.01 level ;*Correlation was significant at the 0.05 level

| **Continent** | **Country** | **Location** | **Time** | **No** | **PFOS** | **PFOA** | **PFHxS** | **Literature** |
| --- | --- | --- | --- | --- | --- | --- | --- | --- |
| Asia | China | 12 provinces | 2006-2008 | 233-blood | 3.06-34.0 | 0.25-3.87 | 0.18-1.40 | 1 |
|  |  | 8provinces | 2004 | 85-blood | 3.72-78.05 | 0.26-1.55 | 0.17-1.91 | 2 |
|  |  | 4provinces | 2004 | 30-blood | 1.4-56.3 | 0.325-1.39 | 0.05-1.87 | 3 |
|  |  | Liaoning^a^ | 2008 | 138-blood | 3.63-7.41 | 0.44-3.81 | 0.61-2.76 | 4 |
|  |  | Hong Kong | 2010-2011 | 153- plasma | 8.68 | 4.02 | 1.34 | 5 |
|  |  | Guangdong | 2012-2013 | 181-serum | 2.094 | 1.85 | 0.344 | our |
|  |  | Guangxi | 2012-2013 | 14-serum | 1.09 | 1.29 | 0.23 | our |
|  |  | Hainan | 2012-2013 | 7-serum | 1.68 | 1.05 | 0.37 | our |
|  | Korea | Daegu | 2003 | 50-blood | 21.1 | 61.8 | 3.95 | 6 |
|  | Japan | Yokohama and Tsukuba | 2002 | 38-sera | 17.1 | 9.55 | 3.75 | 6 |
|  | India | Coimbatore | 2000 | 45-sera | 2 | 3.25 | 1.6 | 6 |
|  | Malaysia | kuala Lumpur | 2004 | 23-blood | 12.45 | <10 | 2.1 | 6 |
| America | United States | / | 2001-2002 | 1832-serum | 20.75 | 3.73 | 2.84 | 7 |
|  | Colombia | Cartagena | 2003 | 56-blood | 8.25 | 6.15 | 0.2 | 6 |
|  | Brazil | Rio Grande | 2003 | 29-blood | 12.1 | <20 | 3.2 | 6 |
| Europe | Italy | Siena | 2001 | 50-sera | 4.35 | <3 | 1.5 | 6 |
|  | Poland | Gdaήsk | 2003 | 25-blood | 44.35 | 21.2 | 1.3 | 6 |
|  | Belgium | Flanders,Wallonia | 1998，2000 | 20-plasma | 13.95 | 4.55 | 1.15 | 6 |
|  | Spain | Catalonia | 2007 | 48-blood | 7.64 | 1.80 | 3.56 | 8 |
|  | Norwegian | / | 2004 | 315-plasma | 22 | 5.1 | 1.4 | 9 |
| Oceania | Australia | Queensland | 2006-2007 | 2420-blood | 15.2 | 6.4 | 3.1 | 10 |

**Table S5.** Perfluorochemical mean concentrations in serum or blood samples (ng/mL) from various countries. a: GM Concentrations

|  | **PFOS** | **PFOA** | **PFHxS** | **PFPrA** | **PFPeA** | **PFBA** | **PFHxA** | **PFBS** |
| --- | --- | --- | --- | --- | --- | --- | --- | --- |
| PFOA | 0.51** |  |  |  |  |  |  |  |
| PFHxS | 0.65** | 0.53** |  |  |  |  |  |  |
| PFPrA | 0.11 | 0.03 | -0.03 |  |  |  |  |  |
| PFPeA | 0.10 | 0.24** | -0.04 | 0.35** |  |  |  |  |
| PFBA | -0.05 | -0.05 | -0.12 | 0.51** | 0.38** |  |  |  |
| PFHxA | 0.17* | 0.31** | 0.04 | 0.17* | 0.51** | 0.31** |  |  |
| PFBS | -0.07 | 0.20** | 0.13 | 0.11 | -0.02 | 0.09 | 0.12 |  |
| ∑_8_PFASs | 0.86** | 0.80** | 0.69** | 0.21** | 0.27** | 0.04 | 0.30** | 0.13 |

**Table S6.** Spearmen’s rank correlation coefficients between PFASs concentrations.*Correlation was significant at the 0.05 level(two-tailed); **Correlation was significant at the 0.01 level(two-tailed)

**Instrument conditions**

Chromatography was performed on a Zorbax Eclipse plus C18 column (Agilent, 3.5 µm, 3.0 × 150 mm) and a C18 guard column (Agilent, 5 µm, 2.1 × 12.5 mm) with gradient elution. An extra trap column (Agilent C18, 2.7 µm, 3.0 × 50 mm) was inserted between the pump and injector to trap contaminants originating from the LC system. The limit of detection (LOD) and quantitation (LOQ) were determined with a signal-to-noise ratio of 3 and 10. The LOD for PFOS and PFOA was 0.001 and 0.15 ng/mL respectively, and for other PFASs it ranged from 0.001 to 0.07 ng/mL.

| **Analytes** | **CAS** | **Brand** | **MRM (CE)** | **DP** | **EP** | **CXP** | **CAD** | **HPLC conditions (0.4mL/min)** | | |
| --- | --- | --- | --- | --- | --- | --- | --- | --- | --- | --- |
| PFOS | 1763-23-1 | Dr.Ehrenstorfer | 499.1/79.5 (98)、98.7 (86) | 20 | 4 | 9 | 9 | Time | 0.1% formic acid | Methanol (0.1%formic acid) |
| PFBA | 375-22-4 | Damas-beta | 212.9/168.5 (13)、62.9 (27) | 23 | 5 | 7 | 8 | 0 | 35 | 65 |
| PFOA | 335-67-1 | AccuStandard | 413.0/368.8 (16)、168.5 (25) | 20 | 7 | 12 | 8 | 7 | 35 | 65 |
| PFBS | 29420-49-3 | Damas-beta | 298.9/79.6 (65)、98.6 (53) | 41 | 5 | 9 | 9 | 7.1 | 5 | 95 |
| PFHxS | 3871-99-6 | Sigma-Aldrich | 398.9/79.7 (88)、98.8 (70) | 6 | 3 | 12 | 9 | 14 | 5 | 95 |
| PFPrA | 422-64-0 | Alfa Aesar | 162.9/118.3 (14)、68.7 (47) | 5 | 6 | 15 | 9 | 14.1 | 35 | 65 |
| PFHxA | 307-24-4 | Tokyo Chemical Industry | 313.1/268.5 (13)、118.8 (29) | 32 | 7 | 10 | 6 | 19 | 35 | 65 |
| PFPeA | 2706-90-3 | Tokyo Chemical Industry | 263.0/218.4 (12)、62.5 (29) | 6 | 6 | 6 | 9 | **MS conditions**  Curtain Gas:30; Collision Gas:50; Ionspray Voltage:-4500V;  Temperature:600℃; Ion Source GAS:60; | | |
| 13C4-PFOA | / | Wellington Laboratories | 416.9/371.8(14)、168.4(25) | 20 | 10 | 14 | 6 |
| 13C4-PFOS | / | Wellington Laboratories | 502.8/79.7 (95)、98.8 (95) | 130 | 10 | 9 | 6 |

**Table S7.** HPLC-MS-MS paramaters and Information of PFASs


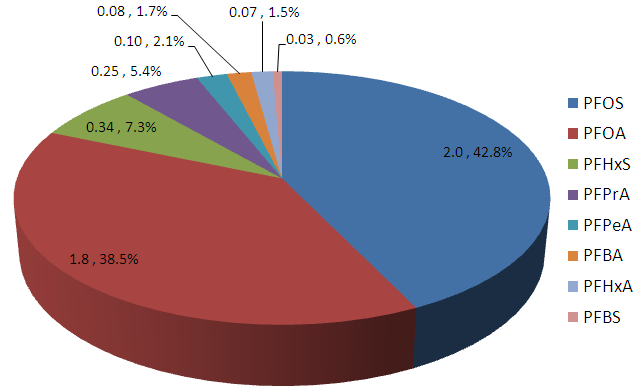


**Figure S1.** Composition profiles of PFASs in serum from the southern Chinese general population

**Figure S2.** Line chart of the mean concentrations of PFASs in different age group

**
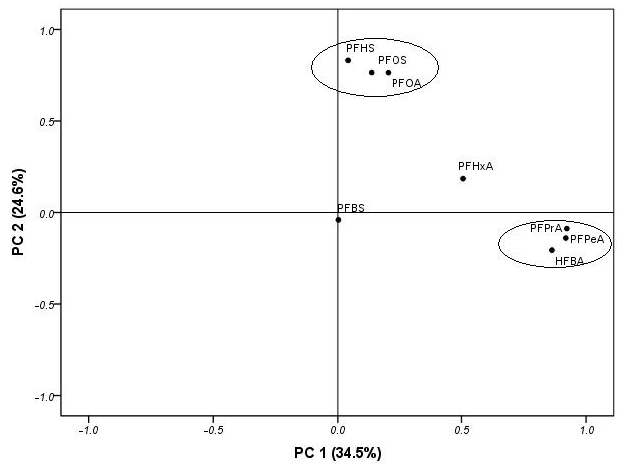
**

**Figure S3.** Plot of factors of principal component analysis of PFASs in serums among the southern Chinese general population

**References**

1. Pan, Y.; Shi, Y.; Wang, J.; Cai, Y.; Wu, Y. Concentrations of perfluorinated compounds in human blood from twelve cities in China*. Environ. Toxicol. Chem***. 2**9, 2695-2701 (2010).

2. Yeung, L.W.Y., et al. Perfluorooctanesulfonate and related fluorochemicals in human blood samples from China*. Environmental science & technolog***y 4**0, 715-720 (2006).

3. Yeung, L.W.Y., et al. Perfluorinated compounds and total and extractable organic fluorine in human blood samples from China*. Environmental science & technolog***y 4**2, 8140-8145 (2008).

4. Liu, J.; Li, J.; Luan, Y.; Zhao, Y.; Wu, Y. Geographical distribution of perfluorinated compounds in human blood from Liaoning province, China*. Environmental science & technolog***y 4**3, 4044-4048 (2009).

5. Wan, H.T., et al. Blood plasma concentrations of endocrine disrupting chemicals in Hong Kong populations*. J. Hazard. Mater***. 26**1, 763-769 (2013).

6. Kannan, K., et al. Perfluorooctanesulfonate and related fluorochemicals in human blood from several countries*. Environmental science & technolog***y 3**8, 4489-4495 (2004).

7. Calafat, A.M.; Kuklenyik, Z.; Caudill, S.P.; Reidy, J.A.; Needham, L.L. Perfluorochemicals in pooled serum samples from United States residents in 2001 and 2002*. Environmental science & technolog***y 4**0, 2128-2134 (2006).

8. Ericson, I., et al. Perfluorinated chemicals in blood of residents in Catalonia (Spain) in relation to age and gender: A pilot study*. Environment Internationa***l 3**3, 616-623 (2007).

9. Rylander, C.; Sandanger, T.M.; Frøyland, L.; Lund, E. Dietary Patterns and Plasma

Concentrations of Perfluorinated Compounds in 315 Norwegian Women: The

NOWAC Postgenome Study. *Environmental Science & Technology* **44,** 5225-5232

(2010).

10. Toms, L.-M.L., et al. Polyfluoroalkyl Chemicals in Pooled Blood Serum from Infants, Children, and Adults in Australia*. Environmental Science & Technolog***y 4**3, 4194-4199 (2009).
